# Use of high frequency oscillatory ventilator in neonates with respiratory failure: the clinical practice in Taiwan and early multimodal outcome prediction

**DOI:** 10.1038/s41598-020-63655-8

**Published:** 2020-04-20

**Authors:** Mei-Chin Yang, Jen-Fu Hsu, Hsiu-Feng Hsiao, Lan-Yan Yang, Yu-Bin Pan, Mei-Yin Lai, Shih-Ming Chu, Hsuan-Rong Huang, Ming-Chou Chiang, Ren-Huei Fu, Ming-Horng Tsai

**Affiliations:** 1 Department of Respiratory Therapy, Chang Gung Memorial Hospital, Taipei, Taiwan; 2 Division of Neonatology, Department of Pediatrics, Chang Gung Memorial Hospital, Taoyuan, Taiwan; 3 School of Business, Executive MBA program in Health Care Management, Chang Gung University, Taoyuan, Taiwan; 4 Division of Neonatology and Pediatric Hematology/Oncology, Department of Pediatrics, Chang Gung Memorial Hospital, Yunlin, Taiwan; 5 College of Medicine, Chang Gung University, Taoyuan, Taiwan; 6 Biostatistics Unit of Clinical Trial Center, Chang Gung Memorial Hospital, Linkou, Taiwan

**Keywords:** Diseases, Molecular medicine, Risk factors, Signs and symptoms

## Abstract

High-frequency oscillatory ventilation (HFOV) can be a rescue for neonates with refractory respiratory failure or an early elective therapy for preterm infants with severe respiratory distress syndrome (RDS). However, little is known about the current evolution and therapeutic limitations of HFOV. We therefore aimed to describe its use in clinical practice and predict the risk of mortality for neonates receiving HFOV. A retrospective observational study of all neonates treated with HFOV in a quaternary referral NICU between January 2007 and December 2016 was conducted. We classified these patients into five subgroups based on primary respiratory diagnoses. We performed the logistic regression and decision tree regression analyses to identify independent factors of 30-day mortality following HFOV. A total of 1125 patients who were ever supported on HFOV were enrolled, of whom 64.1% received HFOV as a rescue therapy, 27.2% received it as an elective therapy, and 8.7% received it for air leak. An average oxygenation index (OI) greater than 25 in the first 24 hours after the initiation of HFOV and patients with secondary pulmonary hypertension were found to have the greatest risk of in-hospital mortality (p < 0.0001). The overall in-hospital mortality rate was 25.8% (290/1125). Decision tree regression analysis revealed that neonates with refractory respiratory failure who had a pre-HFOV OI value higher than 20.5 and OI values higher than 21.5, 23.5 and 34 at 2 hours, 6 hours, and 12 hours after the use of HFOV, respectively, had a significantly increased risk of 30-day mortality. We identified the predictors and cutoff points of OI before and after the initiation of HFOV in neonates with respiratory failure, which can be clinically used as a reference for 30-day mortality. Further efforts are still needed to optimize the outcomes.

## Introduction

High-frequency oscillatory ventilation (HFOV) is a form of mechanical ventilation that can reduce ventilator-associated lung injury, achieve adequate alveolar ventilation with small tidal volumes, and be used as a rescue or early elective therapy for protecting immature lungs^1,2^. Several recently published studies have demonstrated that HFOV can improve oxygenation efficiently and decrease the mortality risk of critically ill patients with acute respiratory distress syndrome, and it has very minimal hemodynamic side effects^3–5^. In the neonatal intensive care unit (NICU), the early use of HFOV has been suggested to be a safer and more effective rescue strategy for neonates with meconium aspiratory syndrome, congenital diaphragmatic hernia, severe pulmonary hypertension, or air leak syndrome^6–11^.

In recent years, whether elective HFOV is more beneficial and effective than conventional ventilation in preterm infants has been highly debated issue and a central topic of many systemic reviews, meta-analyses and randomized controlled trials^12–15^. Cools *et al*. concluded that HFOV seems more effective across various subpopulations of preterm infants^12,13^, and HFOV has been increasingly used in NICUs for patients who do not respond to or tolerate conventional ventilation. However, few studies have broadly reflected the practices and outcomes of neonates who receive HFOV, and there is a paucity of data regarding the risk factors of mortality or treatment failure. Therefore, the main objective of the present study was to describe the evolution of HFOV clinical practice in the NICU over the past 10 years as well as the predictive determinants of 30-day mortality after neonates receiving HFOV.

## Materials and Methods

From January 2007 to December 2016, we conducted a retrospective analysis of observational studies using data prospectively entered into the NICU database^16,17^ of Chang Gung Memorial Hospital (CGMH), a university-affiliated teaching hospital in northern Taiwan. The database is fed by a dedicated nurse specialist and contains all information of patient demographics, clinical features and diagnoses, therapeutic agents and nosocomial infections. The respiratory therapists also maintain all records of daily ventilatory settings in this database. During the study period, all NICU patients with respiratory failure treated by HFOV with/without iNO were enrolled for analysis in this study. This study was approved by the Institutional Review Board (IRB) of Chang Gung Memorial Hospital (CGMH) (IRB approval number: CGMH 103-5758B), and the need for informed consent was waived because all patient records/information were anonymized and deidentified prior to analysis. All methods were performed in accordance with the relevant guidelines and regulations.

In our NICU, the treatment of respiratory distress begins with conventional ventilation (Babylog 8000; Dräger, Lübeck, Germany) and high fractions of inspired oxygen (FiO_2_) to maintain postductal arterial oxygen saturation ≥95% (for cyanotic heart disease, ≥75%) and partial arterial oxygen pressure (PaO_2_) > 60–80 mmHg, depending on the gestational age of the neonates^18^. Patients are sedated with fentanyl and/or midazolam and, if needed, given a muscle relaxant. In newborns with systemic hypotension, the cardiovascular inotropic agents administered include dopamine and dobutamine (both maximum 20 μg/kg/min), milrinone (maximum 1 μg/kg/min) and/or epinephrine (maximum 1 μg/kg/min)^18^. For persistent pulmonary hypertension of newborn (PPHN), iNO (Solmix 1000; Dutch Technical Gas Company, Tilburg, The Netherlands) is added from 20 ppm to a maximum of 80 ppm, based on the clinical responses^18,19^. Surfactant (beractant, Sruventa; Ross Laboratories, Columbus, PH; one dose = 100 mg/kg) is administered to newborns with meconium aspiration syndrome (MAS) and respiratory distress syndrome (RDS). Finally, HFOV (Loudspeaker type, 3100 A; SensorMedics Corporation, Yorba Linda, CA) is used when conventional ventilation fails to improve oxygenation and/or in the presence of severe hypercapnia or barotraumas^18,19^.

### Data collection and definitions

All patients receiving HFOV during the study period were reviewed, and the following information was collected and recorded on the case report form: the etiology of respiratory distress, underlying comorbidities, conventional ventilator management prior to and after HFOV, arterial blood gas data, ventilation mode and ventilator settings on HFOV for the first 72 hours, use of iNO, surfactant, inotropic and vasopressor agents and patient outcomes. FiO_2_ values and ventilator settings at the time of each blood gas determination were recorded by the respiratory therapists. The corresponding AaDO_2_ was calculated using the formula AaDO_2_ = (Patm − 47) × FiO_2_ − (PaO_2_ + PaCO_2_)^9^. For patients with respiratory failure treated by HFOV more than once during hospitalization, the second HFOV run was considered an independent event if the patient was successfully weaned after the first HFOV event and the second event occurred more than 30 days after the first event. For final outcome analyses, the episode of more severe respiratory failure (based on higher initial OI) was considered for analysis.

The use of HFOV as a rescue therapy was considered for neonates who had OI > 20 before or immediately after HFOV and/or those who showed a poor response to conventional ventilation. Otherwise, the use of HFOV was considered an early elective therapy. Poor response to conventional ventilation was defined as the failure to decrease PCO_2_ by >10% and/or FiO_2_ by >20% within 1 hour of conventional ventilation^18^. Patients and events were stratified into the following five distinct categories based upon gestational age, primary diagnoses and acute treatment failure:

1. Term and late-preterm infants (≥34 weeks) with severe acute respiratory failure (patient did not respond to conventional ventilation).
2. Rescue therapy for premature infants (<34 weeks) with refractory hypoxemia.
3. Early elective use of HFOV: HFOV was used as the main method of ventilation in neonates during the early course of acute respiratory distress, without refractory hypoxemia.
4. Air leak, including pneumothorax, pneumomediastinum and pulmonary interstitial emphysema.
5. Underlying chronic pulmonary comorbidities (severe bronchopulmonary dysplasia with secondary persistent pulmonary hypertension).

We defined bronchopulmonary dysplasia (BPD) based on the diagnostic criteria of the American Thoracic Society^20^. Treatment response was defined as follows: (1) good response: successfully treatment by HFOV, and the modality is weaned or ceased at ≦72 hours; (2) partial response: improvement to a certain extent, but HFOV is still needed beyond 72 hours; (3) partial response and then failure: improvement to a certain extent, but HFOV is still needed beyond 72 hours and followed by treatment failure; and (4) failure: failure to improve on HFOV, followed by patient death or subsequent ECMO (extracorporeal membrane oxygenation) within 72 hours.

### Statistical analysis

Disease groups were compared using the Mann-Whitney *U* test, with the data presented as the median and interquartile range for gestational age, birth weight, conventional ventilation duration and follow-up data. Parameters of conventional ventilations immediately preceding HFOV, such as positive end-expiratory pressure (PEEP), pH, FiO_2_, PaO_2_, oxygenation index (OI), and at initiation of HFOV (such as delta P, power, frequency, and bias flow), are presented as the mean and standard deviation. Groups were compared by analysis of variance with post hoc Dunnett *t* tests with the elective HFOV use group as the reference category^21^. Independent predictors of 30-day mortality risk based on continuous covariates such as pH, AaDO_2_ and OI at initial HFOV were modeled using multivariate logistic regression with the backward selection procedure and the likelihood ratio test to assess significance. The probability of mortality risk using maximum likelihood estimation was derived for each disease group based on a range of OI, adjusted for gestational age and birth weight^22^.

We used a minimal p-value approach for cutoff optimization of OI parameters^23^ at four different time points, including pre-HFOV and 2 hours, 6 hours, and 12 hours after initiation of HFOV use. Univariate and multivariate logistic regression models with forward selection procedures were used to identify independent predictors of 30-day mortality. The area under the receiver operating characteristic curve (ROC) based on the optimized OI values was applied to evaluate and compare the predictive ability of OI parameters for 30-day mortality risk. Statistical analysis was performed using SPSS (version 21.0; IBM, Armonk, NY). All *p*-values were two tailed, and *p*-values < 0.05 were considered to be statistically significant.

### Ethics approval and consent to participate

This study was approved by the Institutional Review Board of CGMH, and the need for informed consent was waived because all patient records/information were anonymized and deidentified prior to analysis.

## Results

A total of 1136 neonates were managed with HFOV during the study period. A total of 11 cases were excluded due to missing outcome data and/or ventilatory data. Therefore, a total of 1125 patients were analyzed in this study. All patients were categorized into one of five predetermined categories based on their primary diagnoses (Table 1), which was determined based on physicians’ documentation in the medical records. The most common use of HFOV in our NICU was rescue therapy for preterm infants with refractory failure (45.1%). Of these patients, 64.1% received HFOV as a rescue therapy, 27.2% received it as an elective therapy, and 8.7% received it for air leak.

**Table 1:** Patient demographics stratified into five subgroups.

Variable	Term and late-preterm infants with acute respiratory failure	Rescue therapy for premature infants	Elective use of HFOV	Air leak	Patients with secondary pulmonary hypertension
No. of patients (%)	161 (14.3)	507 (45.1)	306 (27.2)	98 (8.7)	53 (4.7)
Gestational age (wks)	37.0 (34.6–38.4)	27.0 (25.0–29.0)	26.6 (25.3–28.5)	30.0 (26.4–36.4)	27.1 (25.1-29.0)
Birth body weight (g)	2785 (2410–3100)	855 (680–1150)	845.5 (703–1060)	1222.5 (832–2628.5)	846 (695–1057)
Gender (male/female)	108(67.1)/53(32.9)	288(56.8)/219(43.2)	183(59.8)/123(40.2)	66(67.3)/32(32.7)	36(67.9)/17(32.1)
**Apgar score**					
At 1 min	7.0 (5.0–8.0)	4.0 (3.0–6.0)	5.0 (3.0–7.0)	6.0 (3.0–8.0)	5.0 (3.0–6.5)
At 5 min	9.0 (7.0–9.0)	7.0 (5.0–8.0)	7.0 (6.0–8.0)	8.0 (6.0–9.0)	7.0 (6.0–8.0)
Main respiratory disease*					
RDS	65 (40.4)	375 (74.0)	239 (78.1)	50 (51.0)	0 (0)
PPHN	77 (47.8)	83 (16.4)	14 (4.6)	24 (24.5)	7 (13.2)
MAS	23 (14.3)	3 (0.2)	3 (1.0)	6 (6.1)	0 (0)
Pneumonia	12 (7.5)	11 (2.2)	5 (1.6)	0 (0)	3 (5.7)
CDH	9 (5.6)	7 (1.4)	2 (0.7)	1 (1.0)	0 (0)
Sepsis	28 (17.4)	99 (19.5)	29 (9.5)	6 (6.1)	31 (58.5)
Pneumothorax	3 (1.9)	15 (3.0)	8 (2.6)	79 (80.6)	1 (1.9)
Secondary PH	1 (0.6)	2 (0.4)	0 (0)	0 (0)	53 (100)
Pul. hemorrhage	16 (9.9)	60 (11.8)	11 (3.6)	8 (8.2)	3 (5.7)
BPD	2 (1.2)	14 (2.8)	17 (5.6)	2 (2.0)	53 (100)
PDA	85 (52.8)	229 (45.2)	152 (49.7)	30 (30.6)	4 (7.6)
IVH (≥grade III)	6 (3.7)	53 (10.5)	20 (6.5)	4 (4.1)	2 (3.8)
Hydrops Fetalis	11 (6.8)	7 (1.4)	1 (0.3)	6 (6.1)	1 (1.9)
Congenital anomalies^#^	8 (5.0)	3 (0.6)	1 (0.3)	1 (1.0)	2 (3.8)
Congenital heart disease	13 (8.1)	12 (2.4)	0 (0)	0 (0)	0 (0)

Data are presented as number (percentage) or median (interquartile range).

*Indicates concurrent respiratory diseases and disease entities which occurred at initiation of HFOV treatment.

^#^Includes esophageal atresia with tracheo-esophageal fistula (7), cleft palate (3), Pierre Robin syndrome (1), pulmonary sequestration (4).

RDS: respiratory distress syndrome; PPHN: persistent pulmonary hypertension of newborn; MAS: meconium aspiration syndrome; CDH: congenital diaphragmatic hernia; Secondary PH: secondary pulmonary hypertension; BPD: bronchopulmonary dysplasia; PDA: patent ductus arteriosus; IVH: intraventricular hemorrhage.

For the entire cohort, the median (IQR) gestational age and birth weight were 27.6 (25.4–32.0) weeks and 965.5 (740–1600) g, respectively. A total of 74.3% of the patients were less than 32 weeks of gestational age, and 72.8% were very low birth weight (VLBW, birth weight <1500 g) infants. Among the 1125 patients, 86.3% of all HFOV treatments were initiated within the first week of life, and 66.4% of the infants received HFOV starting on their first day of life. A total of 89 (7.9%) patients received a 2^nd^ HFOV treatment after successful weaning from their first use and at least 14 days after their first use due to pneumothorax (1.4%, n = 16), severe sepsis (1.8%, n = 20) or severe BPD with secondary hypertension (4.3%, n = 48). The median duration of HFOV use was 5.0 (3.0–16.0) days, and the median (IQR) duration of intubation with mechanical ventilation was 23.0 (8.0–53.0) days.

### Initiation of HFOV

Table 2 presents the conventional ventilation settings just prior to the initiation of HFOV. Term and preterm infants with acute respiratory failure and patients with chronic lung disease and secondary pulmonary hypertension had a significantly higher AaDO_2_, FiO_2_, mean airway pressure (MAP), and oxygenation index than the other groups (all p < 0.05) prior to initiation of HFOV. In the group of term and preterm infants with acute respiratory failure and patients with secondary hypertension, more than 70% of patients had an OI greater than 15 preceding initiation of HFOV. Inhaled nitric oxide (iNO) and surfactant were used in 18.3% and 62.9% of cases, respectively. Cardiac inotropic agents were used in 69.1% of all events, and nearly half (49.2%) of them required more than one cardiac inotropic agent.

**Table 2:** Baseline characteristics and conventional ventilation setting immediately preceding high-frequency oscillatory ventilation (HFOV).

Variable	Term and late-preterm infants with acute respiratory failure	Rescue therapy for premature infants	Elective use of HFOV	Air leak	Patients with secondary pulmonary hypertension
**Conventional ventilation**					
Peak inspiratory pressure (cm H_2_O)	18.0 (16.5–22.0)*	15.0 (14.5–17.5)	15.0 (14.0–17.5)	16.5 (14.0–18.0)	18.0 (15.0–23.0)*
Mean airway pressure (cm H_2_O)	10.0 (9.0–12.0)*	9.0 (8.0–10.0)	8.5 (8.0–9.3)	9.0 (7.5–10.5)	12.0 (10.0–14.0)**
Respiratory rate/min	45.0 (40.0–50.0)	48.0 (42.0–50.0)	44.0 (39.0–48.0)	45.0 (39.0–46.0)	50.0 (42.0–52.0)*
FiO_2_	95.0 (60.0–100.0)**	65.0 (50.0–90.0)	45.0 (36.0–55.0)	65.0 (50.0–80.0)	84.0 (60.0–100.0)**
PH	7.18 (6.87–7.27)*	7.22 (7.06–7.32)	7.29 (7.22–7.34)	7.22 (7.13–7.29)	7.31 (7.19–7.37)
PaCO_2_, mmHg	48.8 (36.8–61.1)	57.0 (49.0–68.0)*	47.6 (39.6–56.5)	52.0 (42.0–61.0)	63.0 (50.4–72.0)**
PaO_2_, mmHg	42.5 (24.5–55.5)**	51.5 (38.5–67.5)	54.0 (41.5–73.3)	49.5 (39.5–64.5)	48.0 (38.0–52.5)*
Oxygenation index	20.0 (12.0–31.5)*	12.0 (7.0–20.0)	6.0 (4.0–8.0)	12.0 (7.0–24.0)	23.0 (14.0–31.0)**
Baseline AaDO_2_	455.0 (333.0–584.0)*	329.0 (222.0–479.0)	161.0 (108.5–225.3)	293.5 (159.0–461.0)	453.0 (258.0–568.0)*
**Baseline characteristics**^**¶**^					
iNO use	70 (43.5)*	79 (15.6)	19 (6.2)	19 (19.4)	19 (35.8)*
Surfactant use	86 (53.4)	359 (70.8)	214 (69.9)	47 (48.0)	2 (3.8)**
Dopamine	147 (91.3)**	370 (73.0)*	166 (54.2)	66 (67.3)	28 (52.8)
Dobutamine	91 (56.5)*	188 (37.1)	59 (19.3)	28 (28.6)	16 (30.2)
Epinephrine	22 (13.7)	49 (9.7)	8 (2.6)	6 (6.1)	7 (13.2)
Milrinone	24 (14.9)	28 (5.5)	4 (1.3)	7 (7.2)	11 (20.8)*

Data are presented as the median (interquartile range) for conventional ventilation settings.

^¶^Data are presented as number (percentage).

All values were compared with each other with *P < 0.05 and **P < 0.001 by *x*2 test after Bonferroni correction.

### Response to HFOV

Table 3 presents the initial HFOV settings for each of the disease categories. For the entire cohort, the MAP was increased from 9.3 ± 4.7 cm H_2_O on conventional ventilation to 12.7 ± 8.6 cm H_2_O at initiation of HFOV. The OI, FiO_2_, and AaDO_2_ at initiation of HFOV were also significantly higher than those on conventional ventilation (all *p* < 0.001). More than 70% of patients in the category of term and late-preterm infants with acute respiratory failure and those with secondary hypertension had an OI greater than 20.

**Table 3:** Initial high-frequency oscillatory ventilation settings.

Variable	Term and late-preterm infants with acute respiratory failure	Rescue therapy for premature infants	Elective use of HFOV	Air leak	Patients with secondary pulmonary hypertension
Days of starting HFOV, median (IQR)	1.0 (1.0–2.0)	1.0 (1.0–4.0)	1.0 (1.0–3.0)	1.0 (1.0–2.0)	56.0 (32.0–100.5)
DeltaP, cm H_2_O	30.0 (22.5–36.5)	100.0 (77.5–100)	100 (100–100)	51.5 (26.0–100.0)	40.0 (31.0–85.0)
Frequency, Hz	12.0 (11.0–13.0)	12.0 (11.0–13.0)	13.0 (11.0–14.0)	13.0 (11.0–14.0)	12.0 (10.5–13.0)
FiO_2_	100 (80–100)	90 (80–100)	45.0 (40.0–56.3)	77.5 (51.5–100)	100.0 (75.0–100.0)
Oxygenation index	25.0 (17.5–39.0)	15.0 (10.0–28.0)	6.0 (4.0–9.0)	15.0 (10.0–30.0)	29.0 (22.0–46.8)
AaDO_2_	528.0 (391.5–590.0)	393.0 (250.0–565.0)	167.0 (111.3–234.0)	367.0 (211–579)	556.0 (378.0–595.5)
Mean airway pressure (cmH_2_O)	15.0 (13.0–17.0)	11.0 (10.0–13.0)	10.0 (8.0–11.0)	11.0 (10.0–14.0)	16.0 (13.0–20.0)
PH*	7.23 (7.14–7.30)	7.24 (7.11–7.35)	7.32 (7.26–7.38)	7.26 (7.13–7.36)	7.35 (7.27–7.44)
PaO_2_	51.6 (36.0–70.9)	52.9 (36.5–79.7)	71.9 (52.9–98.3)	56.9 (38.9–80.1)	46.7 (37.3–59.3)
PaCO_2_	52.9 (41.8–67.3)	52.0 (42.3–66.8)	44.2 (37.2–51.1)	54.9 (43.3–63.9)	52.0 (44.3–70.0)

Data are presented as the median (interquartile range).

*Some data may be checked after sodium bicarbonate replacement.

To assess changes in HFOV and the therapeutic response to HFOV, we examined the OI at 2 hours, 6 hours and 12 hours after initiation of HFOV and the average daily OI, AaDO2, and blood gas analysis on the 2^nd^ and 3^rd^ day after use of HFOV. Table 4 shows the treatment outcomes after the use of HFOV. A total of 755 (67.1%) patients showed a good response to HFOV and could be weaned within 3 days, whereas 87 (7.7%) patients died due to refractory respiratory failure within 3 days after the initiation of HFOV. Patients with severe BPD and secondary pulmonary hypertension had the highest risk of treatment failure and in-hospital mortality (both p < 0.001).

**Table 4:** Treatment outcomes of neonates treated by high-frequency oscillatory ventilation (HFOV).

Treatment outcomes	Term and late-preterm infants with acute respiratory failure	Rescue therapy for premature infants	Elective use of HFOV	Air leak	Patients with secondary pulmonary hypertension
Good response	94 (58.4)	314 (61.9)	276 (90.2)	61 (62.2)	10 (18.9)
Partial response	28 (17.4)	71 (14.0)	15 (4.9)	13 (13.3)	16 (30.2)
Partial response and then failure	7 (4.3)	36 (7.1)	9 (2.9)	5 (5.1)	7 (13.2)
Failure	32 (19.9)	86 (17.0)	6 (2.0)	19 (19.4)	20 (37.7)
Progress to BPD*	17/154 (11.0)	252/443 (56.9)	191/276 (69.2)	32/95 (33.7)	—
Duration of HFOV (days)	4.0 (2.0–6.0)	5.0 (3.0–19.0)	8.0 (3.0–27.0)	4.5 (3.0–8.0)	9.0 (3.5–28.5)
Duration of intubation (days)	5.0 (2.0–10.0)	23.0 (4.0–50.0)	32.0 (10.8–54.3)	7.0 (3.5–39.0)	57.0 (32.5–91.3)
Duration of mechanical ventilation (days)	9.0 (6.0–17.0)	40.0 (12.0–73.0)	53.0 (24.0–75.3)	10.0 (5.0–54.0)	76.0 (43.5–132.0)
30-day mortality	40 (24.8)	154 (30.4)	35 (11.4)	24 (24.5)	31 (58.5)

Data are presented as the number (percentage), while duration of HFOV, intubation and mechanical ventilation are expressed as median (interquartile range).

BPD: bronchopulmonary dysplasia, defined based on the diagnostic criteria of the American Thoracic Society^21^.

*Only considers the patients who received HFOV during the first two weeks of life and survived for more than 4 weeks.

### Quantification of 30-day mortality risk and treatment failure

When all categories were analyzed, 290 patients died and the in-hospital mortality rate was 25.8% (290/1125). Patients with secondary pulmonary hypertension showed the highest mortality risk (58.5%, p < 0.001 when compared with other subgroups). All potential parameters of the respiratory ventilations were first analyzed to find the highest predictive power of treatment failure. According to the multivariate logistic regression analysis, an average oxygenation index (OI) greater than 25 within the first 24 hours after initiation of HFOV and patients with secondary pulmonary hypertension exhibited the greatest predictive power (p < 0.0001) for increased mortality risk.

**Figure 1:**
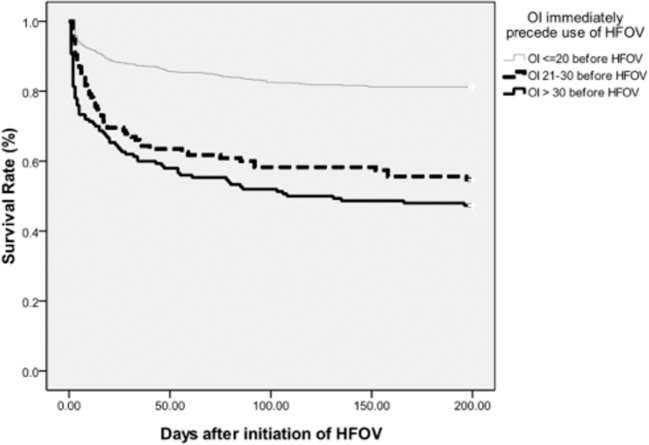
Survival following the initiation of HFOV in neonates from the neonatal intensive care unit of CGMH 2010–2017. The Kaplan-Meier survival graph is stratified by the OI immediately preceding the use of HFOV, which showed that OI > 20.5 had a significantly higher rate of mortality (OI: oxygenation index, HFOV: high frequency oscillatory ventilation).

**Figure 2:**
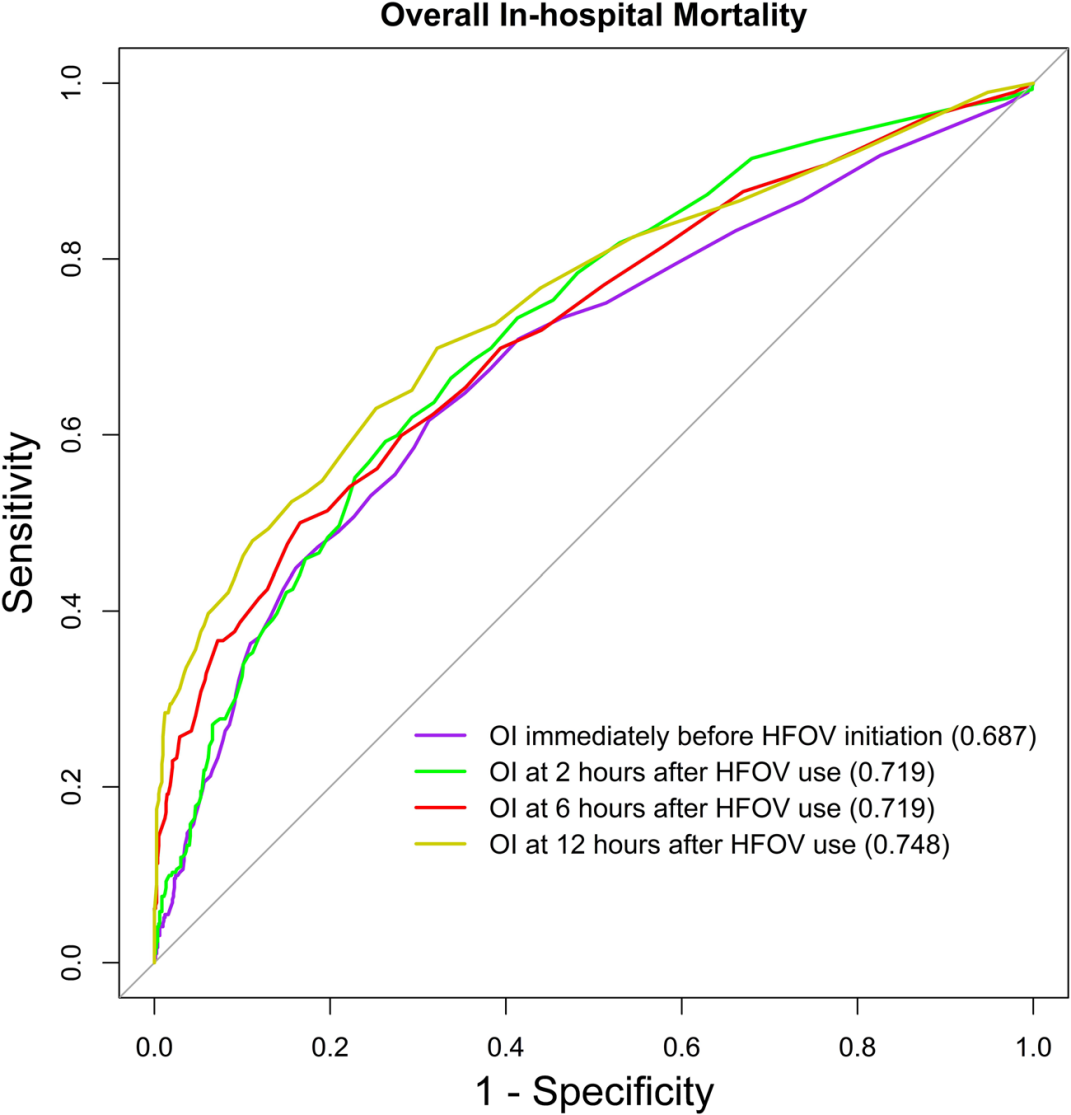
The receiver-operator characteristic (ROC) curve of the predictive cutoff points based on OIs at four different time points; the areas under the curve are 0.687, 0.719, 0.719 and 0.748 for these four time points.

We used decision tree regression to identify the minimal p value in order to determine the cutoff point with the highest predictive power for early prediction of 30-day mortality. The pre-HFOV OI and the OIs on the first day at three different points after the use of HFOV were evaluated. We found that an OI of 20.5 immediately preceding the use of HFOV, an OI of 21.5 within 2 hours after initiation of HFOV, an OI greater than 23.5 at 6 hours and an OI greater than 34.5 at 12 hours after initiation of HFOV had the highest predictive power of 30-day mortality. For example, the Kaplan-Meier graph is stratified by the OI immediately preceding the use of HFOV, which showed that an OI > 20.5 had a significantly higher rate of mortality (Fig. 1). The receiver-operator characteristic (ROC) plot of these predictive cutoff points is shown in Fig. 2, and the areas under the curve are 0.687, 0.719, 0.719 and 0.748 for these four time points.

## Discussion

The indication and selection of neonates with respiratory failure for HFOV use in the NICU is not well defined^13^. Although HFOV seems to be a promising technique for rescue therapy and decreases mortality in preterm infants with refractory respiratory failure, there is little literature regarding its therapeutic limit and predictors of treatment failure. In the current study, we found that neonates with secondary pulmonary hypertension had the highest mortality risk, and the probability of 30-day mortality risk could be estimated by computing the OIs on conventional ventilations before initiation of HFOV and 2, 6, and 12 hours after use of HFOV. These results indicated that the treatment outcomes can be predicted early by the OI cutoff points, which represented the initial severity of respiratory failure and the initial therapeutic response of HFOV use^24^.

In addition to elective use of HFOV, 84% of patients with acute respiratory failure had an OI greater than 16, and 48% had an OI greater than 24, which indicated the rescue strategy in our NICU. We also found that the OIs recorded after the initiation of HFOV were always higher than those recorded on conventional ventilation preceding HFOV, which was consistent with other studies^1,24^. Therefore, we suspect that the data on conventional ventilation immediately preceding the use of HFOV cannot reflect the real indication of HFOV use. It is possible that adverse hemodynamic effects from pulmonary diseases, hypoxemia, hypercarbia or a combination of these factors make these conditions continuously worsen. It takes some time for the therapeutic effects of HFOV to reverse hypoxemia or hypercarbia. In addition, some neonates in the subgroup of elective HFOV use experienced deterioration while receiving HFOV treatment and 11.4% in this group ultimately died. It is possible that these patients eventually required rescue HFOV support or ECMO. We did not use conventional ventilation until maximal support, and those without OIs greater than 16 could be considered as preemptive use but not a primary mode of ventilation.

The mortality rate of our cohort treated with HFOV was high and did not show a trend of improvement. However, not all patients died of refractory hypoxemia^19,24^. In our institute, we always use HFOV as the final rescue therapy for critically ill neonates with cardiopulmonary failure. These patients contributed to a significant proportion of mortality, which could mask the therapeutic effects of HFOV. Most of the patients died of underlying diseases or secondary nosocomial infections instead of hypoxemia and pulmonary diseases. The area under the curve in the ROC plot was between 0.687 and 0.748, which are acceptable but not very powerful values. Neonates with a hemodynamically stable condition and good response to the use of HFOV may have later died of sepsis or other chronic comorbidities. Therefore, it is difficult to have highly distinctive OI cutoff points with a better predictive value.

In our cohort treated with HFOV, premature infants with RDS accounted for approximately one-third of the events. Previous studies have found that preterm infants with severe respiratory failure who received initial ventilation with HFOV could have a lower risk of death and BPD and have superior long-term pulmonary function and neurodevelopmental outcomes^25–27^. A randomized control trial even documented that the first-intension use of HFOV could reduce the need for ventilatory support and reintubation and shorten NICU stays and costs in VLBW infants^28^. Therefore, although the use of HFOV is not financial, a comparison of elective HFOV with conventional ventilation in extremely low birth weight infants is warranted in the future^12–14,27,28^.

Nearly one-fifth of the patients (18.3%, n = 206) received iNO, mostly due to PPHN (71.4%), and some of them were extremely preterm infants with severe RDS (19.4%). Another 19 cases (9.2%) were treated due to secondary pulmonary hypertension. iNO is effective in term infants with hypoxic respiratory failure^29^, but recent studies have concluded that iNO does not appear to be as effective as rescue therapy for very ill preterm infants^30,31^. iNO can improve the oxygenation of premature infants without PPHN, although its effectiveness should be further studied^30–32^. Both the American Academy of Pediatrics and National Institutes of Health (NIH) have expressed concern that the use of iNO therapy is extremely low birth weight infants could increase the risk of IVH^33,34^. Despite this safety concern, the off-label use of iNO in extremely hypoxemic preterm infants on maximal ventilator support has been investigated recently, and the beneficial effects were found to outweigh the disadvantages without an increased risk of IVH^18,35^.

A total of 8.7% patients who received HFOV had persistent air leakage. Although HFOV is not a standard treatment for neonates with air leak, it can have beneficial effects on the prevention of recurrent pneumothorax and reverse severe hypercapnia^36,37^. For low birth weight infants, the lower mean frequency of our preterm infants under HFOV (12~13) is due to the ventilator machine, with Babylog 8000 (Dräger, Lübeck, Germany) switched from conventional mode to HFOV mode. Under this machine, a lower mean frequency is capable of preventing or reversing CO2 retention. It should be noted that HFOV is not routinely used at our institute in neonates with air leak. We categorized these patients because the air leak worsened their underlying pulmonary diseases in most situations and HFOV became the rescue strategy.

This study has several limitations that need to be addressed. First, the initiation of HFOV was based on the attending physician’s decision, and the indication to change from conventional ventilation to HFOV is not yet standardized. Because there is no control group and we did not compare our study subjects with those without the HFOV treatment, we can not comment on the beneficial effects or disadvantages of HFOV over conventional ventilation. Second, this is a single retrospective study. Some of the parameters were not controlled and may be adjusted after some treatments. Finally, categorizing these patients into distinct disease subgroups may have led to some ambiguity, which may have resulted in statistical deviations. However, the strengths of this study include the large sample size, the prospectively collected database with detailed and accurate parameters of ventilatory settings at various time points and the concisely defined responses. Therefore, the predictive OI cutoff points at four stages are convincing and can be clinically used as a reference for clinicians to explain patient prognosis in discussions with family members.

## Data Availability

The datasets used/or analyzed during the current study are available from the corresponding author upon reasonable request.
